# Amelioration of Japanese encephalitis by blockage of 4-1BB signaling is coupled to divergent enhancement of type I/II IFN responses and Ly-6C^hi^ monocyte differentiation

**DOI:** 10.1186/s12974-015-0438-x

**Published:** 2015-11-24

**Authors:** Seong Bum Kim, Jin Young Choi, Jin Hyoung Kim, Erdenebelig Uyangaa, Ajit Mahadev Patil, Sang-Youel Park, John Hwa Lee, Koanhoi Kim, Young Woo Han, Seong Kug Eo

**Affiliations:** College of Veterinary Medicine and Bio-Safety Research Institute, Chonbuk National University, Iksan, 54596 Republic of Korea; Department of Bioactive Material Sciences, Graduate School, Chonbuk National University, Jeonju, 54896 Republic of Korea; Department of Pharmacology, School of Medicine, Pusan National University, Yangsan, 50612 Republic of Korea

**Keywords:** 4-1BB signal, Japanese encephalitis, Type I/II IFN, Ly-6C^hi^ monocytes, Zoonotic diseases, Neurologic disorder

## Abstract

**Background:**

Japanese encephalitis (JE), a neuroinflammation caused by zoonotic JE virus, is the major cause of viral encephalitis worldwide and poses an increasing threat to global health and welfare. To date, however, there has been no report describing the regulation of JE progression using immunomodulatory tools for developing therapeutic strategies. We tested whether blocking the 4-1BB signaling pathway would regulate JE progression using murine JE model.

**Methods:**

Infected wild-type and 4-1BB-knockout (KO) mice were examined daily for mortality and clinical signs, and neuroinflammation in the CNS was evaluated by infiltration of inflammatory leukocytes and cytokine expression. In addition, viral burden, JEV-specific T cell, and type I/II IFN (IFN-I/II) innate responses were analyzed.

**Results:**

Blocking the 4-1BB signaling pathway significantly increased resistance to JE and reduced viral burden in extraneural tissues and the CNS, rather than causing a detrimental effect. In addition, treatment with 4-1BB agonistic antibody exacerbated JE. Furthermore, JE amelioration and reduction of viral burden by blocking the 4-1BB signaling pathway were associated with an increased frequency of IFN-II-producing NK and CD4^+^ Th1 cells as well as increased infiltration of mature Ly-6C^hi^ monocytes in the inflamed CNS. More interestingly, DCs and macrophages derived from 4-1BB KO mice showed potent and rapid IFN-I innate immune responses upon JEV infection, which was coupled to strong induction of PRRs (RIG-I, MDA5), transcription factors (IRF7), and antiviral ISG genes (ISG49, ISG54, ISG56). Further, the ablation of 4-1BB signaling enhanced IFN-I innate responses in neuron cells, which likely regulated viral spread in the CNS. Finally, we confirmed that blocking the 4-1BB signaling pathway in myeloid cells derived from hematopoietic stem cells (HSCs) played a dominant role in ameliorating JE. In support of this finding, HSC-derived leukocytes played a dominant role in generating the IFN-I innate responses in the host.

**Conclusions:**

Blocking the 4-1BB signaling pathway ameliorates JE via divergent enhancement of IFN-II-producing NK and CD4^+^ Th1 cells and mature Ly-6C^hi^ monocyte infiltration, as well as an IFN-I innate response of myeloid-derived cells. Therefore, regulation of the 4-1BB signaling pathway with antibodies or inhibitors could be a valuable therapeutic strategy for the treatment of JE.

## Background

The incidence of zoonotic diseases transmitted to humans from wild or domestic animals has increased noticeably during the past few decades and currently represents at least 70 % of emerging diseases [1]. Japanese encephalitis virus (JEV), a zoonotic, mosquito-borne *Flavivirus*, is considered to be a major cause of viral encephalitis worldwide. Due to rapid changes in climate and demography, vector-transmitted JEV poses an increasing threat to global health and welfare with approximately 67,900 cases reported annually, despite large, effective immunization campaigns [2–4]. The incubation period of JE ranges from 5 to 15 days and JEV infections are lethal in about 25–30 % of cases, mostly in infants, and cause permanent neuropsychiatric sequelae in 50 % of cases [4]. Accordingly, JE is considered to be more fatal than West Nile (WN) encephalitis, which results in a fatality in 3–5 % of cases (1100 deaths/29,000 symptomatic infections) [5]. Currently, more than 60 % of the world’s population inhabits JE endemic areas, including eastern and southern Asia, and the virus is spreading to previously unaffected regions, such as Indonesia, Pakistan, and northern Australia [2, 3].

JE is a neuroinflammation characterized by extensive CNS inflammation and disruption of the blood–brain barrier (BBB) after zoonotic JEV infection. Considerable progress in understanding the kinetics and mechanisms of JEV dissemination and JE pathogenesis has been made in murine models [6–8]. However, the molecular pathogenesis of JE remains elusive. After peripheral introduction of the virus via mosquito bites, JEV initially replicates in primary target cells, such as dendritic cells (DCs) and macrophages, at the periphery and subsequently gains entry into the CNS through the BBB. While JEV infects and kills neurons directly in the CNS, CNS invasion by JEV also drives the stimulation of microglia/glia and infiltrated leukocytes, leading to indirect death of neuron cells via secretion of pro-inflammatory cytokines (such as IL-6 and TNF-α) and soluble mediators [9, 10]. Therefore, JE is considered an immunopathological disease in which uncontrolled over-activation of innate and adaptive immune cells drives neurological disorders in the CNS such as paralysis. While JEV-specific T cells and virus-neutralizing IgM and IgG are considered to participate in the clearance of virus from both peripheral lymphoid tissues and the CNS [11], innate immune responses appear to play a more crucial role in the early control of JEV infection, due to delayed establishment of adaptive immunity. The type I IFN (IFN-I; typically IFN-α/β) innate immune response is essential for controlling various viral infections, including JEV [12–15], and IFN-I production is triggered by recognition of viral pathogen-associated molecular patterns (PAMPs) through cytoplasmic helicases (RIG-I, MDA5) and Toll-like receptors (TLRs) [16–20]. In addition, recent data indicate that type II IFN (IFN-II; only member IFN-γ) produced by NK and CD4^+^ Th1 cells has a beneficial effect on disease outcomes after JEV infection [21, 22], although the requirement for IFN-II in recovery from infection with different flaviviruses varies [21, 23–29].

Recently, the debatable role of CD11b^+^Ly-6C^hi^ monocytes in the course of neuroinflammation caused by pathogenic CD4^+^ T cells or neurotropic viruses has initiated a new era in the exploration of their differentiation lineage and immunopathological role in the CNS [30]. These Ly-6C^hi^ monocytes migrate into the infected CNS, where they differentiate into DCs, macrophages, and arguably microglia to regulate neuroinflammation [30–32]. Despite the conflicting results of studies investigating the role of Ly-6C^hi^ monocytes in modulating neuroinflammation, CNS infiltration by CD11b^+^Ly-6C^hi^ monocytes is required for the control of neuroinflammation, which supports their protective role during CNS inflammation [33–36]. Notably, the differentiation levels of Ly-6C^hi^ monocytes that infiltrate into the CNS appear to affect the progression of neuroinflammation caused by various insults [37–39]. However, restraint of CNS infiltration of leukocytes from the periphery, including innate and adaptive immune cells, is also required because hematogenous inflammation causes profound damage if the reaction is excessive or uncontrollable [40]. Therefore, a clear understanding of the regulation of excessive and uncontrollable immune responses during JE progression is needed to ameliorate the progression of neuroinflammation without tissue damage.

4-1BB (CD137) is a member of the tumor necrosis factor receptor (TNFR) superfamily, and its role as a T cell co-stimulatory molecule has been well defined [41]. However, 4-1BB molecules are also expressed on a variety of innate immune cells, including NK cells, DCs, monocytes, and neutrophils [42–46]. Stimulation of the 4-1BB signal is believed to enhance protective immune responses against pathogens, because agonistic anti-4-1BB mAbs can enhance the efficacy of vaccines against influenza and poxvirus [47, 48]. The importance of the 4-1BB receptor-ligand system in viral infection control is further supported by the fact that 4-1BB ligand-deficient mice exhibit impaired immunity against lymphocytic choriomeningitis virus (LCMV) [49] and a few influenza virus strains [50, 51]. At the same time, stimulation of the 4-1BB signal pathway with agonistic mAbs also inhibits the development of several autoimmune diseases, including lupus, arthritis, and experimental autoimmune encephalomyelitis [52–54], which indicates that there must be contradictory effects from the stimulation of the 4-1BB receptor-ligand system. Therefore, it is likely that 4-1BB signaling plays different roles depending on the properties of diseases: mild vs. severe forms or pathogenic vs. autoimmunogenic. Despite various and seemingly contradictory roles of the 4-1BB receptor-ligand system in various inflammatory diseases, 4-1BB is still an attractive target for the development of therapeutic strategies for incurable inflammatory diseases. However, no reports have yet shown the regulatory effect of the 4-1BB signaling pathway on neuroinflammation caused by neurotropic viruses such as JEV. In this study, somewhat surprisingly, blocking the 4-1BB signaling pathway ameliorated JE progression, rather than causing detrimental effects. Furthermore, the protective role of blocking the 4-1BB signal against JE was likely mediated by enhanced IFN-I innate immune responses in myeloid-derived and neuron cells, an increased number of IFN-γ-producing NK and CD4^+^ Th1 cells, and early and increased infiltration of mature Ly-6C^hi^ monocytes in the CNS. Therefore, our data provide valuable insight into the regulation of the 4-1BB signaling pathway as a therapeutic target for neuroinflammation caused by infection with flaviviruses such as JEV and West Nile virus (WNV).

## Methods

### Ethics statement

All animal experiments were conducted at Chonbuk National University according to guidelines set by the Institutional Animal Care and Use Committees (IACUC) of Chonbuk National University (http://lac.honamlife.com) and were pre-approved by the Ethical Committee for Animal Experiments of Chonbuk National University (Permission code 2013-0028). The animal research protocol in this study followed the guidelines set up by the nationally recognized Korea Association for Laboratory Animal Sciences (KALAS). All experimental protocols requiring biosafety were approved by Institutional Biosafety Committees (IBC) of Chonbuk National University.

### Animals, cells, and viruses

C57BL/6 (H-2^b^) mice (4–5 weeks old) were purchased from Samtako (O-San, Korea). 4-1BB (H-2^b^) knockout (KO) mice were obtained from Ulsan University. All mice were genotyped and bred in the animal facilities of Chonbuk National University. JEV Beijing-1 strain was obtained from the Green Cross Research Institute (Suwon, Korea) and propagated in the mosquito cell line C6/36 using DMEM supplemented with 2 % FBS, penicillin (100 U/ml), and streptomycin (100 U/ml). C6/36 cultures were infected with JEV Beijing-1 at a multiplicity of infection (MOI) of 0.1 and were incubated in a humidified CO_2_ incubator for 1 h at 28 °C. After absorption, the inoculum was removed, and 7 ml of a maintenance medium containing 2 % FBS was added. Approximately 6–7 days post-infection (dpi), cultures of host cells showing an 80–90 % cytopathic effect were harvested. Virus stocks were titrated using either a conventional plaque assay or a focus-forming assay and were stored in aliquots at −80 °C until use.

### Antibodies and reagents

The following mAbs used for flow cytometric analysis and other experiments were obtained from eBioscience (San Diego, CA, USA) or R&D Systems (Minneapolis, MN, USA): fluorescein isothiocyanate (FITC) conjugated-anti-CD4 (RM4-5), CD8 (53-6.7), CD40 (HM40-3), CD44 (IM7), CD80 (16-10A1), CD86 (GL1), F4/80 (8 M8), MHC I (28-14-8), MHC II (M5/114.15.2), and Ly-6G (1A8); phycoerythrin (PE) conjugated-anti-mouse-CD11b (M1/70), Foxp3 (FJK-16 s), CD154(MR1), CCR2 (475301), CXCR2 (242216), and granzyme B (NGZB); peridinin chlorophyll protein complex (PerCP) conjugate-anti-Ly-6C (HK1.4); PE-Cyanine dye (Cy5.5)-anti-mouse IFN-γ (XMG1.2); PE-Cyanine dye (Cy7)-anti-mouse NK1.1 (PK136); and allophycocyanin (APC) conjugated-anti-mouse-CD25 (PC62.5), Ly-6G (Gr-1), TNF-α (MP6-XT22), and IL-17A (eBio17B7). Peptides of I-A^b^-restricted epitopes (JEV NS1_132–145_ [TFVVDGPETKECPD] and NS3_563–574_ [WCFDGPRTNAIL]) and H-2D^b^-restricted epitope (JEV NS4B_215–223_ [SAVWNSTTA]) were chemically synthesized at Peptron Inc. (Daejeon, Korea). JEV-specific primers for the detection of viral RNA (JEV10,564-10,583 forward, 5′-CCC TCA GAA CCG TCT CGG AA-3′ and JEV10,862-10,886 reverse, 5′-CTA TTC CCA GGT GTC AAT ATG CTG T-3′) and primers specific for cytokines, type I IFNs (IFN-α/β), and RLRs, IRFs, ISGs (Table 1) were synthesized at Bioneer Corp. (Daejeon, Korea) and used for PCR amplification of target genes.

**Table 1:** Specific primers for cytokine, type I IFNs, PRRs, IRFs, and ISGs used in real-time qRT-PCR

Gene name^a^	Primer sequence (5′-3′)^b^	Position cDNA	Gene Bank ID
TNF-α	FP: CGT CGT AGC AAA CCA CCA AG	438-457	NM_013693
	RP: TTG AAG AGA ACC TGG GAG TAG ACA	564-587	
IFN-α	FP: TGTCTGATGCAGCAGGTGG	367-385	NM_008334.3
	RP: AAGACAGGGCTCTCCAGAC	514-532	
IFN-β	FP: TCCAAGAAAGGACGAACATTCG	106-121	NM_010510
	RP: TGAGGACATCTCCCACGTCAA	399-419	
IRF3	FP: GAT GGA GAG GTC CAC AAG GA	1170-1189	NM_016849
	RP: GAG TGT AGC GTG GGG AGT GT	1259-1278	
IRF7	FP: CCT CTT GCT TCA GGT TCT GC	980-999	NM_016850.3
	RP: GCT GCA TAG GGT TCC TCG TA	1080-1099	
RIG-I	FP: CCA CCT ACA TCC TCA GCT ACA TGA	194-217	NM_172689
	RP: TGG GCC CTT GTT GTT CTT CT	260-279	
MDA5	FP: GGC ACC ATG GGA AGT GAT T	1178-1196	NM_027835
	RP: ATT TGG TAA GGC CTG AGC TG	1247-1266	
ISG49	FP: GCC GTT ACA GGG AAA TAC TGG	919-939	NM_010501.2
	RP: CCT CAA CAT CGG GGC TCT	1126-1143	
ISG54	FP: GGG AAA GCA GAG GAA ATC AA	1918-1937	NM_008332.3
	RP: TGA AAG TTG CCA TAC AGA AG	2005-2024	
ISG56	FP: CAG AAG CAC ACA TTG AAG AA	774-793	NM_008331.3
	RP: TGT AAG TAG CCA GAG GAA GG	911-930	
β-Actin	FP: TGG AAT CCC TGT GGG ACC ATG AAA C	885-909	NM_007393.3
	RP: TAA AAC GCA GCT CAG TAA CAG TCC G	1209-1233	

^a^ *IL* interleukin, *TNF-α* tumor necrosis factor-α, *IFN* interferon

^b^ *FP* forward primer, *RP* reverse primer

### Quantitative real-time RT-PCR for viral burden and cytokine expression

Viral burden and cytokine (TNF-α, IFN-α, and IFN-β) expression in inflammatory and lymphoid tissues were determined by conducting quantitative SYBR Green-based real-time RT-PCR (real-time qRT-PCR). Mice were infected intraperitoneally (i.p.) with JEV (3.0 × 10^7^ PFU) and tissues including the brain, spinal cord, and spleen were harvested at 2, 4, and 6 dpi following extensive cardiac perfusion with Hanks balanced salt solution (HBSS). Total RNA was extracted from tissues using easyBLUE (iNtRON, INC., Daejeon, Korea) and subjected to real-time qRT-PCR using a CFX96 Real-Time PCR Detection system (Bio-Rad Laboratories, Hercules, CA, USA). Following reverse transcription of total RNA with High-Capacity cDNA Reverse Transcription Kits (Applied Biosystems, Foster, CA, USA), the reaction mixture contained 2 μl of template cDNA, 10 μl of 2× SYBR Primix Ex Taq, and 200 nM primers for a final volume of 20 μl. The reactions were denatured at 95 °C for 30 s and then subjected to 45 cycles of 95 °C for 5 s and 60 °C for 20 s. After the reaction cycle was complete, the temperature was increased from 65 to 95 °C at a rate of 0.2 °C/15 s, and the fluorescence was measured every 5 s to construct a melting curve. A control sample that contained no template DNA was run with each assay, and all determinations were performed at least in duplicate to ensure reproducibility. The authenticity of the amplified product was determined by melting curve analysis. All data were analyzed using the Bio-Rad CFX Manager, version 2.1 analysis software (Bio-Rad Laboratories).

### Analysis and activation of NK cells

The activation of NK cells was assessed by the capacity to produce IFN-γ and granzyme B (GrB) following brief stimulation with PMA and ionomycin (Sigma-Aldrich). Splenocytes were prepared from BL/6 and 4-1BB KO mice 2 dpi and stimulated with PMA (50 ng/ml) and ionomycin (750 ng/ml) in the presence of monensin (2 μM) to induce the expression of IFN-γ and GrB for 1 and 2 h, respectively. After stimulation, cells were surface stained by FITC anti-mouse-CD3ε, PE-Cy7 anti-mouse NK1.1, and biotin-conjugated anti-mouse pan-NK cell (CD49b) [DX5] antibodies and streptavidin-APC for 30 min at 4 °C. The cells were then washed twice with FACs buffer containing monensin. After fixation, cells were permeabilized with 1× permeabilization buffer (eBioscience) and stained intracellularly with PE anti-mouse IFN-γ (XMF1.2) and GrB antibodies (NGZB) in permeabilization buffer for 30 min at 4 °C. Finally, the cells were washed with PBS twice, and analysis was performed with FACS Calibur flow cytometer (Becton Dickson Medical Systems, Sharon, MA, USA) and FlowJo software (ver. 7.6.5; Tree Star, San Carlos, CA, USA).

### JEV-specific CD4^+^ and CD8^+^ T cell responses

JEV-specific CD4^+^ and CD8^+^ T cell responses were determined by intracellular CD154 [55, 56] as well as IFN-γ and TNF-α staining in response to stimulation with respective JEV epitope peptides. Surviving mice infected with JEV (3.0 × 10^7^ PFU) were sacrificed at 7 or 14 dpi, and splenocytes were prepared. The erythrocytes were depleted by treating single-cell suspensions with ammonium chloride-containing Tris buffer (NH_4_Cl-Tris) for 5 min at 37 °C. The splenocytes were cultured in 96-well culture plates (5 × 10^5^ cells/well) in the presence of synthetic peptide epitopes (NS1_132–145_, NS3_563–574_, and NS4B_215–225_) for 12 and 6 h, in order to observe CD4^+^ and CD8^+^ T cell responses, respectively. Monensin (2 μM) was added to antigen-stimulated cells 6 h before harvest. The cells were washed twice with PBS and surface stained with FITC-anti-CD4 or CD8 antibodies for 30 min at 4 °C and then washed twice with PBS containing monensin again. After fixation, the cells were washed twice with permeabilization buffer (eBioscience) and stained with PE Cy5.5-anti-IFN-γ or APC-anti-TNF-α in permeabilization buffer for 30 min at room temperature. Intracellular CD154 was detected by the addition of CD154 mAb in the culture media during peptide stimulation. Finally, the cells were washed twice with PBS and fixed using a fixation buffer. Sample analysis was performed with FACS Calibur flow cytometer (Becton Dickson Medical Systems) and FlowJo (Tree Star) software.

### Intracellular staining for analysis of CD4^+^ Th1, Th17, and Treg cells

To monitor CD4^+^ Th subsets, mice infected with JEV (3.0 × 10^7^ PFU) were sacrificed at 3 and 5 dpi, and splenocytes were prepared. Splenocytes were cultured in 96-well culture plates (10^6^ cells/well) with PMA (50 ng/ml) plus ionomycin (750 ng/ml) in the presence of monensin (2 μM) for 5 h at 37 °C. The stimulated cells were washed twice with PBS and surface stained with FITC-anti-CD4 for 30 min at 4 °C and then washed twice with PBS containing monensin. After fixation, the cells were washed twice with permeabilization buffer (eBioscience) and stained with PerCP-anti-IFN-γ and APC-anti-IL-17α in permeabilization buffer for 30 min at room temperature. Finally, the cells were washed twice with PBS and fixed using fixation buffer. To monitor Treg cells, splenocytes were surface stained for FITC-anti-CD4 markers for 30 min on ice and then fixed with fixation/permeabilization concentrate buffer (eBioscience) for 6 h at 4 °C. After fixation, the cells were washed twice with permeabilization buffer and stained with PE-anti-Foxp3 in permeabilization buffer for 30 min at room temperature. The sample analysis was performed with FACS Calibur flow cytometer.

### Analysis of leukocytes infiltrated into the CNS

Mice infected with JEV were perfused with 30 ml of HBSS at 2 or 4 dpi via cardiac puncture of the left ventricle. Brains were then harvested and homogenized by gently pressing them through a 100-mesh tissue sieve, after which they were digested with 25 μg/ml of collagenase type IV (Worthington Biochem, Freehold, NJ, USA), 0.1 μg/ml trypsin inhibitor *Nα*-*p*-tosyl-L-lysine chloromethyl ketone, 10 μg/ml DNase I (Amresco, Solon, OH, USA), and 10 mM HEPE in HBSS for 1 h at 37 °C, under shaking conditions. Cells were separated by using an Optiprep density gradient (18/10/5 %) centrifugation at 800 × g for 30 min (Axis-Shield, Oslo, Norway), after which cells were collected from the 18 to 10 % interface and washed twice with PBS. Cells were counted and stained for CD11b, Ly6G, Ly6C, F4/80, and MHC II with directly conjugated antibodies (eBioscience) for 30 min at 4 °C. Finally, the cells were fixed with 10 % formaldehyde. Data collection and analysis were performed with FACS Calibur flow cytometer (Becton Dickson Medical Systems) and FlowJo (Tree Star) software.

### Primary cell culture and infection

#### Myeloid-derived DCs and macrophages

Bone-marrow derived DCs (BMDC) and macrophages (BMDM) were prepared from bone marrow cells of 4-1BB KO and WT mice. In order to prepare BMDC, bone marrow cells (3 × 10^5^ cells/ml) from femurs and tibiae were cultured in RPMI 1640 supplemented with 2 ng/ml GM-CSF and 10 ng/ml IL-4. On day 3, another 6 ml of fresh complete medium containing 2 ng/ml GM-CSF and 10 ng/ml IL-4 was added, and half of the medium was changed on day 6. On day 8, non-adherent and loosely adherent DCs were harvested by vigorous pipetting. Cells were then characterized by flow cytometric analysis, which revealed that the culture generally consisted of >85 % CD11c^+^ cells (25 % CD11c^+^CD11b^+^and 65 % CD11c^+^CD8α^+^). BMDM were prepared by culturing bone marrow cells in DMEM supplemented with 30 % L929 cell-conditioned medium (LCCM) as a source of macrophage-colony-stimulating factor (M-CSF). On day 3, another 6 ml of fresh complete medium containing 30 % LCCM was added, and half of the medium was changed on day 6. The cultured cells were harvested following an 8-day incubation and analyzed by flow cytometry. The prepared BMDM were composed of >85 % F4/80^+^ cells that consisted of 99.2 % F4/80^+^CD11b^+^ and ~1 % F4/80^+^CD11c^+^ cells. Prepared BMDC and BMDM were infected with JEV at MOIs of 1.0 and 10 for viral replication and 10 MOI for cytokine expression.

#### Primary cortical neurons

Primary cortical neurons were prepared from 15-day-old embryos. Cortical neurons were seeded in 12-well poly-D-lysine/laminin-coated plates in DMEM containing 5 % FBS and 5 % horse serum for 24 h and then cultured for 4 days with neurobasal medium containing B27 supplement and L-glutamine (Invitrogen, Carlsbad, CA, USA). Primary cortical neurons were infected with JEV at a 0.1 MOI for viral replication and type I IFN responses.

### Generation of BM chimeric mice and determination of serum IFN-β

C57BL/6 mice (5-week-old) and 4-1BB KO mice were γ-irradiated with one dose of 900 rads. Within 12 h, recipient mice were reconstituted with 10^7^ donor BM cells derived from C57BL/6 and 4-1BB KO mice. The recipient mice were given sulfamethoxazole and trimethoprim in their drinking water for 10 days after irradiation. Mice were infected with JEV 4–6 weeks after irradiation. A commercial ELISA kit (PBL Biomedical Laboratories, Piscataway, NJ, USA) was used to measure levels of secreted IFN-β protein in the sera, according to the manufacturer’s protocol.

### Statistical analysis

All data were expressed as the average ± standard deviation, and statistically significant differences between groups were analyzed by unpaired two-tailed Student’s *t* tests for ex vivo experiments and immune cell analysis or ANOVA and post hoc test for multiple comparisons of the mean. The statistical significance of viral burden was evaluated by Mann–Whitney test or unpaired two-tailed Student’s *t* test. Kaplan-Meier survival curves were analyzed with the log-rank test. A *p* value ≤0.05 was considered significant. All data were analyzed using Prism software (GraphPadPrism4, San Diego, CA, USA).

## Results

### Blocking 4-1BB signaling ameliorates JE

**Fig. 1:**
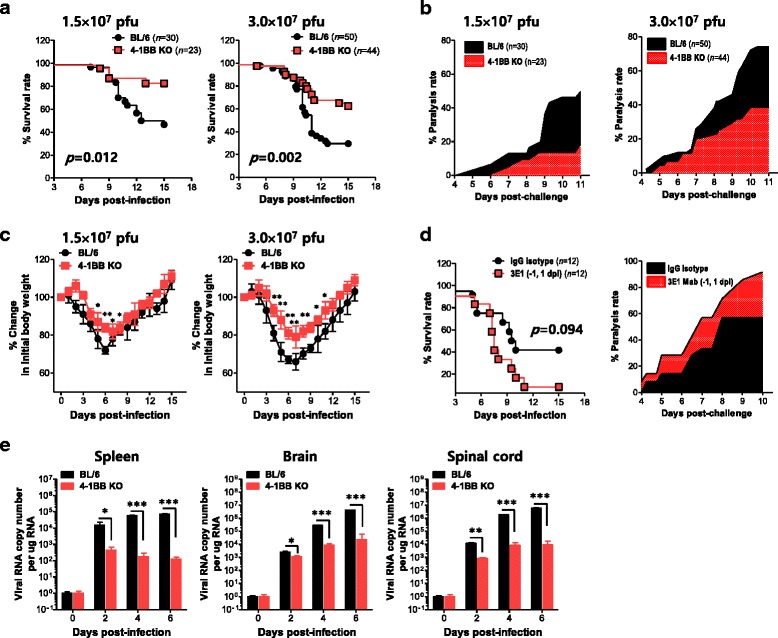
Blocking the 4-1BB signaling pathway ameliorates JE along with a reduction in viral burden. **a** Susceptibility of 4-1BB KO mice to JE. BL/6 and 4-1BB KO mice (4- to 5-week-old, *n* = 23–50) were inoculated i.p. with JEV (1.5 or 3.0 × 10^7^ PFU), and the survival rate was examined over 15 days. **b** Ratio of mice showing neurological disorder during JE progression. Mice infected with JEV were examined every 6 h from 4 to 11 dpi, and the ratio of mice showing neurological disorder in inoculated mice was recorded. **c** Changes in body weight. Changes in body weight are expressed as the average percentage ± SD of body weight relative to the time of challenge. **d** Exacerbation of JE by 4-1BB signal stimulation. WT mice were intravenously given an agonistic antibody (3E1, 400 μg/mouse) to the 4-1BB signal at −1 and 1 dpi and examined for survival and paralysis rate. **e** Viral burden in lymphoid and inflammatory tissues during JE. Viral burden in the spleen, brain, and spinal cord of infected mice was assessed by real-time qRT-PCR at the indicated dpi. The viral RNA load is expressed by viral RNA copy number per microgram of total RNA (*n* = 5–7). *p < 0.05; **p < 0.01; ***p < 0.001 compared with the levels of the indicated groups

Blocking or triggering the 4-1BB signaling pathway has been shown to elicit various and seemingly contradictory results in the regulation of inflammatory diseases, depending on the severity of the disease model [47–54]. Furthermore, the role of the 4-1BB signaling pathway in modulating neuroinflammation caused by neurotropic viruses has been not addressed to date. Therefore, we tested whether blocking 4-1BB signaling would modulate neuroinflammation caused by JEV infection, in order to evaluate the therapeutic potential of the 4-1BB signaling pathway in JE progression. To this end, we examined and compared the susceptibility of 4-1BB-deficient (4-1BB KO) mice to JE caused by neurotropic JEV infection with that of wild-type (WT) BL/6 mice (Fig. 1a). Interestingly, ablation of 4-1BB signaling provided significantly enhanced resistance to JE after infection with two different JEV doses (*p* = 0.012 for 1.5 × 10^7^ and *p* = 0.002 for 3.0 × 10^7^ PFU). Likewise, 4-1BB KO mice showed delayed signs of neurological disorders starting from 5 to 6 dpi, whereas BL/6 mice showed neurological disorders more quickly and at a higher proportion (Fig. 1b). In addition, 4-1BB ablation resulted in less change in body weight (Fig. 1c). These results indicate that ablation of 4-1BB signaling ameliorated JE progression.

This finding prompted us to investigate whether stimulation of the 4-1BB signaling pathway would affect the severity of JE. 3E1 mAb has been used as an agonistic antibody to stimulate 4-1BB signaling in a variety of immune cells, including T cells, dendritic cells (DCs), and NK cells [42–44]. Treatment with agonistic 3E1 mAb significantly reduced the survival of mice with JE and induced faster and enhanced proportions of neurological disorders (Fig. 1d), indicating that stimulating 4-1BB signaling exacerbates JE progression. This result strengthens our finding that blocking 4-1BB signaling provides enhanced resistance to JE. To better understand JE progression in 4-1BB KO mice, we examined the viral burden in peripheral lymphoid and CNS tissues after JEV infection. 4-1BB KO mice were found to contain 10- to 100-fold less virus in the spleen, brain, and spinal cord compared to BL/6 mice (Fig. 1e). Collectively, these results suggest that ablation of 4-1BB signaling ameliorates JE progression by regulating viral burden, while triggering 4-1BB signaling enhances the severity of JE.

### Enhancement of innate NK cell responses in 4-1BB signal-ablated mice

**Fig. 2:**
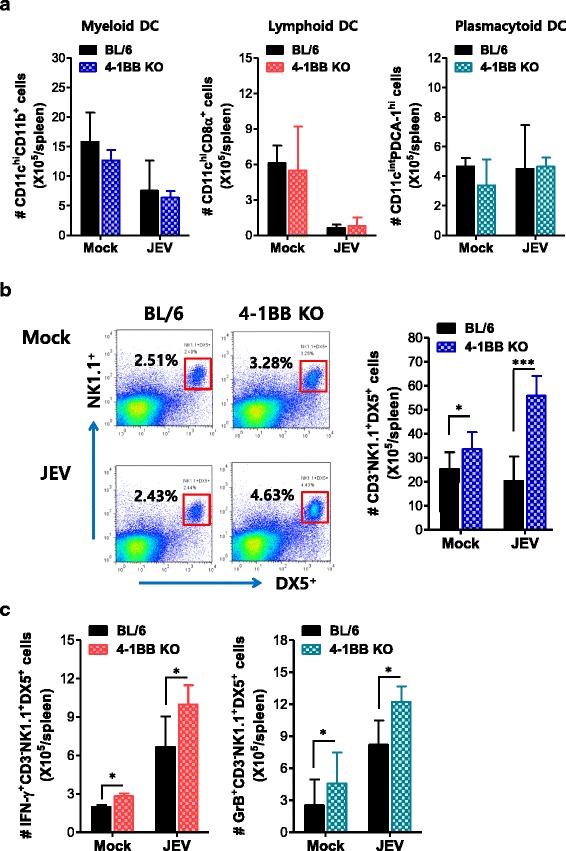
Enhanced development of innate NK cell responses in 4-1BB signal-ablated mice. **a** Absolute number of DC subpopulations. DC subpopulations (myeloid, lymphoid, and plasmacytoid DCs) were enumerated by flow cytometric analysis using collagenase-treated spleen 2 dpi. **b** Frequency and absolute number of NK cells. The frequency and number of CD3^−^NK1.1^+^DX5^+^ NK cells were determined 2 dpi. Values in representative dot-plots denote the average percentage of NK cells after gating on CD3-negative cells. **c** NK cell activation. The activation of NK cells was evaluated by enumerating NK cells producing IFN-γ and granzyme B (GrB) using intracellular cytokine staining 2 dpi. Bar graphs show the average ± SD of values derived from at least four mice per group. *p < 0.05; **p < 0.01; ***p < 0.001 compared with the levels of the indicated groups

4-1BB and its ligand are expressed in innate immune cells as well as BM cells, and attempts to evaluate the regulation of myelopoiesis by the 4-1BB receptor-ligand system have yielded conflicting results [42–54]. Furthermore, signaling in the 4-1BB receptor-ligand system is thought to be bidirectional, where 4-1BB and its ligand are expressed as transmembrane proteins on the cell surface and transmit signals into both cells [57]. Therefore, activation of the 4-1BB ligand in APCs may contribute to the elimination of pathogens by enhancing its APC activity. However, since 4-1BB deficiency may induce the abrogation of 4-1BB ligand signaling during JE progression, JE amelioration in 4-1BB KO mice may occur through other mechanisms. To investigate the mechanisms behind our results, we examined innate immune responses in 4-1BB KO mice upon JEV infection. Our results revealed that JEV infection of 4-1BB KO mice induced no significant alterations in DC subpopulations (CD11c^hi^CD11b^+^ myeloid, CD11c^hi^CD8α^+^ lymphoid, and CD11c^int^PDCA-1^hi^ plasmacytoid DC) (Fig. 2a). However, 4-1BB KO mice showed basally higher frequencies and numbers of CD3^−^NK1.1^+^DX5^+^ NK cells compared to BL/6 mice. Subsequently, the frequencies and number of NK cells were markedly enhanced in 4-1BB KO mice following JEV infection (Fig. 2b), indicating that 4-1BB signaling may interfere with the development of NK cells in wild-type BL/6 mice during JE progression. In addition, 4-1BB KO mice showed enhanced activity of NK cells upon JEV infection when evaluated by enumeration of NK cells producing IFN-γ and granzyme B in response to stimulation of PMA plus ionomycin (Fig. 2c). This result implies that the enhancement of innate NK cell immune responses in 4-1BB KO mice may contribute to increased resistance to JE.

### Impaired T-cell-mediated adaptive immunity in 4-1BB signal-ablated mice following JEV

**Fig. 3:**
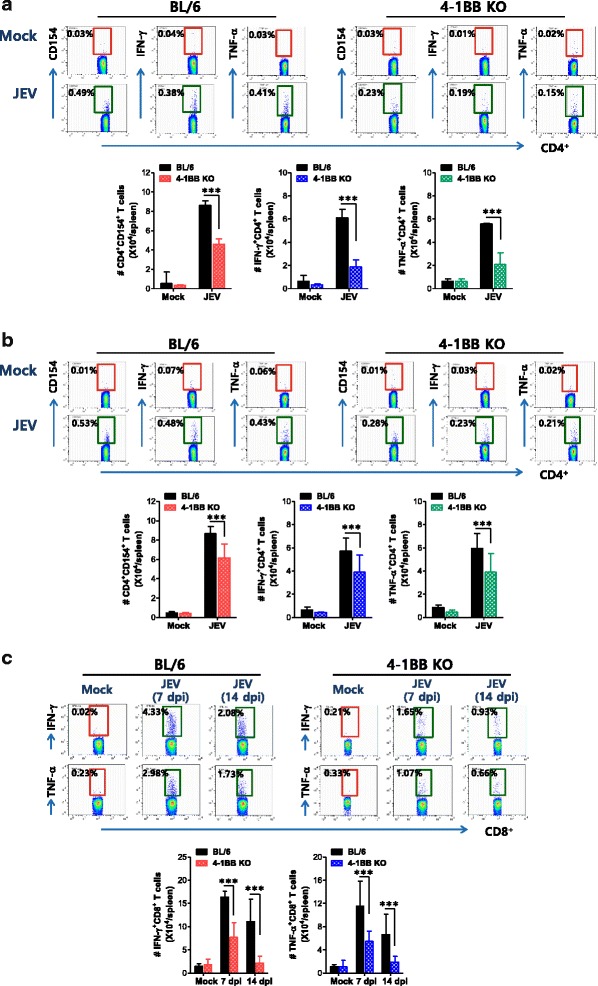
Impaired JEV-specific T cell immunity in 4-1BB signal-ablated mice. **a**, **b** JEV-specific CD4^+^ T cell responses. **c** JEV-specific CD8^+^ T cell responses. Splenocytes prepared from surviving mice at 7 or 14 dpi were stimulated with JEV epitope peptide of CD4^+^ T cells (**a** NS1_132–145_; **b** NS3_563-574_) or CD8^+^ T cells (NS4B_215–223_) for 12 or 6 h, respectively. The frequency and absolute number of JEV-specific CD4^+^ and CD8^+^ T cells were determined by intracellular CD154 and cytokine (IFN-γ and TNF-α) staining, combined with surface CD4 and CD8 staining. Values in representative dot-plots denote the average percentage of the indicated cell population, and bar charts show the average ± SD of values derived from at least four mice per group. *p < 0.05; **p < 0.01; ***p < 0.001 compared with the levels of the indicated groups

Although recent results suggest complex roles for the 4-1BB signal pathway in modulating T cell responses, 4-1BB signaling is considered a positive regulator of T cell responses against pathogens [47, 48]. Therefore, we examined the generation of JEV-specific CD4^+^ and CD8^+^ T cell responses in surviving BL/6 and 4-1BB KO mice at 7 dpi. As expected, our results revealed that ablation of the 4-1BB signal induced a reduction in CD4^+^ T cell responses specific for two epitopes (NS1_132–145_ and NS3_563–574_) derived from JEV Ag (Fig. 3a, b), based on enumeration of JEV-specific CD4^+^ T cells by intracellular CD154 staining [55, 56]. In addition, the total number of CD4^+^ T cells producing IFN-γ and TNF-α in response to epitope peptide stimulation was lower in 4-1BB KO mice than in BL/6 mice. In particular, JEV-specific CD8^+^ T cell responses were 2- to 4-fold lower in 4-1BB KO mice than in BL/6 mice, based on CD8^+^ T cells producing IFN-γ and TNF-α upon stimulation with CD8^+^ T cell epitope (NS4B_215–223_) (Fig. 3c). Collectively, considering that adequate CD4^+^ and CD8^+^ T cell responses may contribute to the regulation of JE progression [21, 22], these results suggest that impaired CD4^+^ and CD8^+^ T cell responses generated in 4-1BB KO mice are not involved in conferring enhanced resistance to JE.

### A skewed response of 4-1BB-ablated mice to IFN-γ-producing CD4^+^ Th1 during JE

**Fig. 4:**
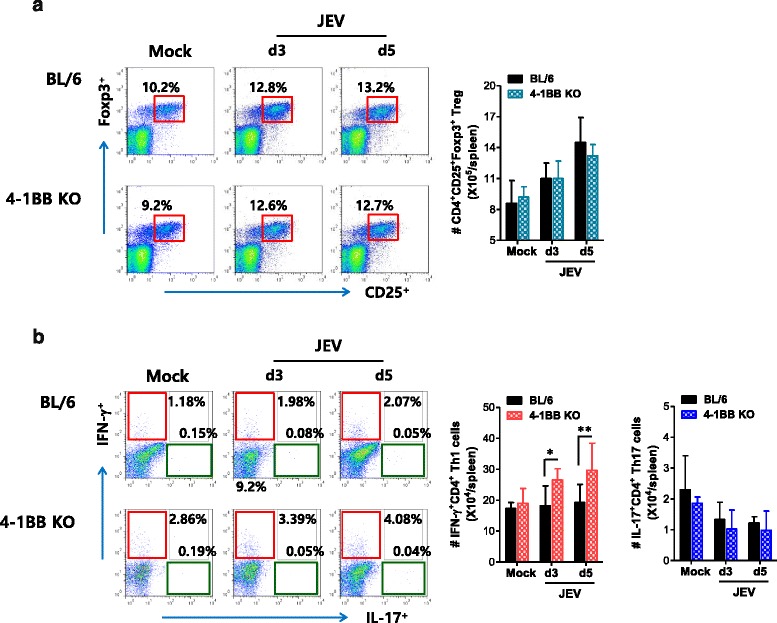
A skewed response of 4-1BB signal-ablated mice to IFN-γ-producing CD4^+^ Th1. **a** The frequency and number of CD4^+^CD25^+^Foxp3^+^ Tregs. The frequency and total number of CD4^+^CD25^+^Foxp3^+^ Treg cells in the spleen of WT and 4-1BB KO mice were determined by flow cytometric analysis 3 and 5 dpi. Dot-plots show the frequency of CD25^+^Foxp3^+^ Tregs after gating on CD4^+^ T cells. **b** The frequency and number of IFN-γ^+^CD4^+^ Th1 and IL-17^+^CD4^+^ Th17 cells. The frequency and number of IFN-γ^+^CD4^+^ Th1 and IL-17^+^CD4^+^ Th17 cells were determined by intracellular cytokine staining in response to PMA + ionomycin stimulation of splenocytes prepared from WT and 4-1BB KO mice 3 and 5 dpi. Values in the representative dot-plots denote the average percentage of the indicated cell populations, and bar graphs show the average ± SD of values derived from at least four mice per group. *p < 0.05; **p < 0.01; ***p < 0.001 compared with the levels of the indicated groups

CD4^+^CD25^+^Foxp3^+^ Treg cells may contribute to the control of neuroinflammation caused by neurotropic viruses [58]. Moreover, 4-1BB signaling has been reported to alter the balance between CD4^+^CD25^+^Foxp3^+^ Treg and IL-17^+^CD4^+^ Th17 cells during inflammatory reactions [59]. In order to investigate additional mechanisms, we also addressed the frequency and number of CD4^+^CD25^+^Foxp3^+^ Treg cells during JE progression. There was no apparent alteration of the number or frequency of CD4^+^CD25^+^Foxp3^+^ Treg cells in 4-1BB KO mice during JE progression (Fig. 4a). However, more CD4^+^ Th1 cells producing IFN-γ were detected in 4-1BB-ablated mice, and decreased frequency and number of IL-17^+^CD4^+^ Th17 cells were observed in both BL/6 and 4-1BB KO mice during JE progression (Fig. 4b). This result indicates that the increase in IFN-γ^+^CD4^+^ Th1 cells, but not CD4^+^CD25^+^Foxp3^+^ Treg or IL-17^+^CD4^+^ Th17 cells, is closely associated with enhanced resistance of 4-1BB KO mice against JE.

### Enhanced CNS infiltration of matured Ly-6C^hi^ monocytes in 4-1BB signal-ablated mice

**Fig. 5:**
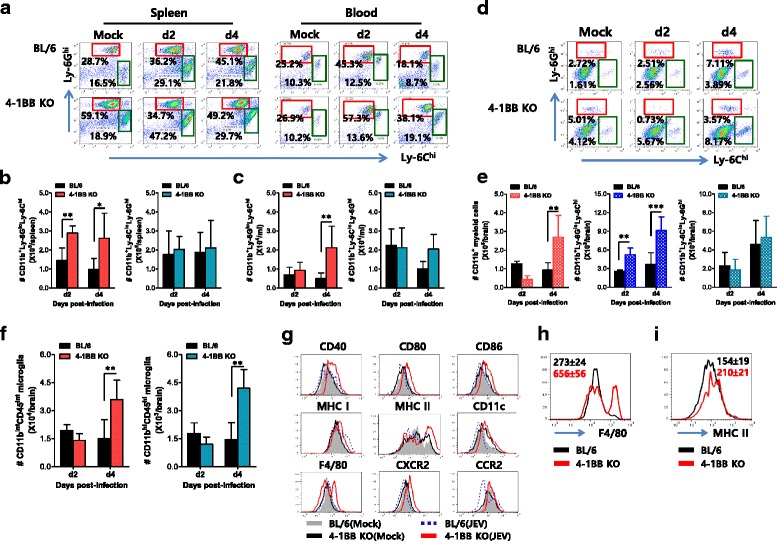
Enhanced infiltration of mature Ly-6C^hi^ monocytes in 4-1BB signal-ablated mice. **a**–**c** The frequency and number of Ly-6C^hi^ monocytes and Ly-6G^hi^ granulocytes in spleen and blood. The frequency (**a**) and total number and of Ly-6C^hi^ monocytes (**b**) and Ly-6G^hi^ granulocytes (**c**) in the spleen and blood were determined by flow cytometric analysis 2 and 4 dpi. Values in representative dot-plots denote the average percentage of the indicated population after gating on CD11b^+^ cells (*n* = 4–5). **d**, **e** Infiltrated Ly-6C^hi^ monocytes and Ly-6G^hi^ granulocytes in the CNS. The frequency (**d**) and number (**e**) of infiltrated Ly-6C^hi^ monocytes and Ly-6G^hi^ granulocytes in the CNS were determined after vigorous heart perfusion. **f** Resting and activated microglia number in the CNS. The number of resting (CD11b^+^CD45^int^) and activated (CD11b^+^CD45^hi^) microglia/macrophage were enumerated by flow cytometric analysis. **g** Phenotypic levels of Ly-6C^hi^ monocytes. The phenotypic levels of infiltrated Ly-6C^hi^ monocytes were determined at 4 dpi after gating on splenic CD11b^+^Ly-6C^hi^ monocytes. **h**, **i** Maturation levels of Ly-6C^hi^ monocytes and microglia. Maturation levels of CNS Ly-6C^hi^ monocytes (**h**) and CD11b^+^CD45^hi^ microglia (**i**) were evaluated by the expression of F4/80 and MHC II, respectively. The MFI in histograms denotes the average ± SD of values derived from at least four mice per group. *p < 0.05; **p < 0.01; ***p < 0.001 compared with the levels of the indicated groups

CNS-infiltration of CD11b^+^Ly-6C^hi^ monocytes is a hallmark of neuroinflammation caused by neurotropic viral infection [30]. Although the role of CD11b^+^Ly-6C^hi^ monocytes is debatable in the progression of neuroinflammation, CNS infiltration and maturation of CD11b^+^Ly-6C^hi^ monocytes are believed to support their protective role during neuroinflammation [33–36]. Accordingly, we examined the frequency and total number of CD11b^+^Ly-6C^hi^ monocytes during JE progression to investigate their role in enhanced resistance of 4-1BB KO mice to JE. Wild-type BL/6 and 4-1BB KO mice showed comparable levels of CD11b^+^Ly-6C^hi^ monocytes in the spleen and blood before JEV infection, but the frequency and absolute number of CD11b^+^Ly-6C^hi^ monocytes increased in the spleen and blood of 4-1BB KO mice during JE progression (Fig. 5a–c). Consistently, 4-1BB KO mice displayed early and increased infiltration of CD11b^+^Ly-6C^hi^ monocytes in the brain following JEV infection, compared to BL/6 mice (Fig. 5d), and the total number of CD11b^+^ myeloid cells and CD11b^+^Ly-6C^hi^ monocytes was also higher in the brain of 4-1BB KO mice (Fig. 5e). However, there were no differences in the total number of CD11b^+^Ly-6G^hi^ granulocytes that had infiltrated into the brain of 4-1BB KO mice compared to BL/6 mice. It has been shown that microglia cells contribute to the pathogenesis of neuroinflammation caused by some neurotropic viruses such as WNV [31]. Thus, triple-color staining (CD11c/CD11b/CD45) was used to distinguish between resting and activated microglia. Based on the CNS myeloid cell classification of Ford et al. [60], the absolute number of both resting (CD11c^−^CD11b^hi^CD45^int^) and activated microglia (CD11c^−^CD11b^hi^CD45^hi^) increased 2- to 3-fold in 4-1BB KO mice compared to BL/6 mice (Fig. 5f).

To further determine whether the maturation of CD11b^+^Ly-6C^hi^ monocytes could be affected by 4-1BB signal ablation, we characterized the phenotypic levels of splenic CD11b^+^Ly-6C^hi^ monocytes. Phenotypic levels of CD11b^+^Ly-6C^hi^ monocytes in uninfected 4-1BB KO mice were not significantly different from those of uninfected BL/6 mice. However, CD11b^+^Ly-6C^hi^ monocytes in the spleen of 4-1BB KO mice displayed more matured phenotypes following JEV infection than did BL/6 mice (Fig. 5g). Notably, F4/80, a phenotypic marker of mature macrophages, was expressed at much higher levels in CD11b^+^Ly-6C^hi^ monocytes that had infiltrated into the brain of 4-1BB KO mice compared to those of BL/6 mice (Fig. 5h). Consistently, activated microglia (CD11c^−^CD11b^hi^CD45^hi^) in the brains of 4-1BB KO mice showed higher expression of MHC II molecules than those of BL/6 mice (Fig. 5i), as one marker of microglia activation [61, 62]. Collectively, these results suggest that early and increased infiltration of mature CD11b^+^Ly-6C^hi^ monocytes in the CNS could be involved in the enhanced resistance of 4-1BB-ablated mice to JE.

### Potent IFN-I innate response of 4-1BB signal-deficient myeloid cells controls JEV replication

**Fig. 6:**
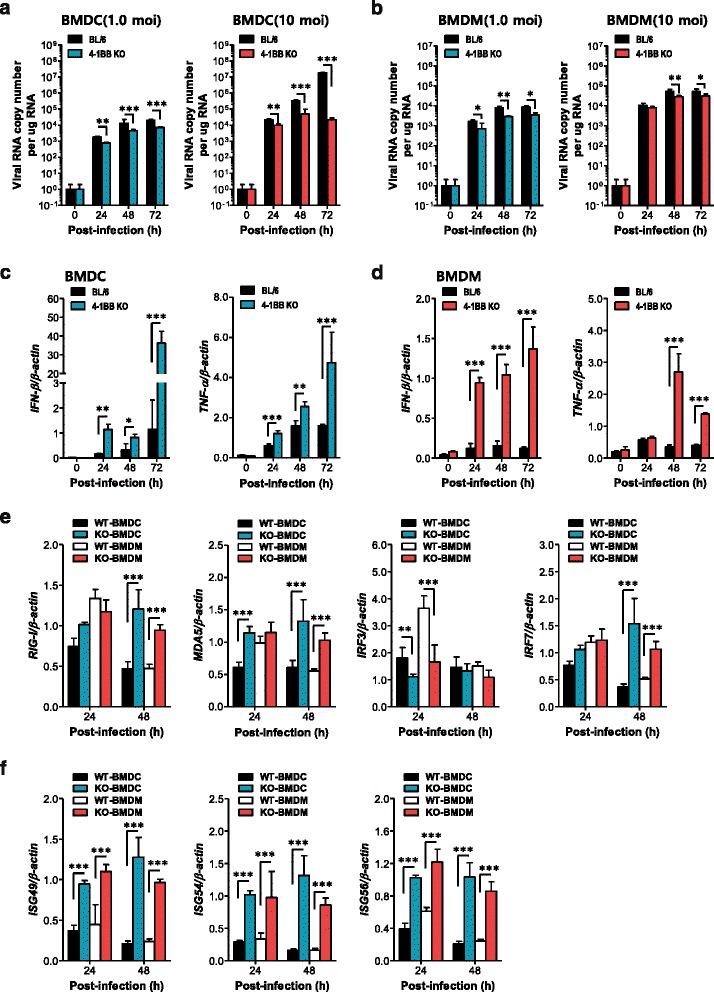
Virus control and IFN-I responses of 4-1BB signal-deficient myeloid cells to JEV infection. Primary bone marrow-derived DCs (BMDC) and macrophages (BMDM) recovered from WT and 4-1BB KO mice were infected with JEV at MOI of 1.0 and 10 for viral replication and 10 for cytokine expression. **a**, **b** JEV replication in BMDC and BMDM. **c**, **d** IFN-β and TNF-α expression in BMDC and BMDM. **e**, **f** Expression of RLR, IRF, and ISG genes in BMDC and BMDM. Bar charts show the average ± SD of values derived from BMDC and BMDM in quadruplicate. *p < 0.05; **p < 0.01; ***p < 0.001 compared with the levels of the indicated groups

Myeloid cells, including both tissue and lymphoid DCs and macrophages, are primary target cells for JEV infection and regulate the spread of a virus to distant tissues such as the CNS [7, 8]. In addition, myeloid cells can produce IFN-I proteins (IFN-α/β) via PRR recognition upon JEV infection, which plays a crucial role in controlling viral replication at the periphery [16–20]. Because virus load at the periphery of 4-1BB KO mice was lower than that of wild-type BL/6 mice in the present study, we assessed whether 4-1BB signaling would affect JEV replication and IFN-I innate response in myeloid-derived cells as primary target cells, in order to further define the role of 4-1BB signaling in controlling JE progression. Bone marrow-derived DCs (BMDC) and macrophages (BMDM) of 4-1BB KO mice were infected with JEV and used to evaluate viral replication and the induction of IFN-I and pro-inflammatory cytokines. Interestingly, 4-1BB-deficient BMDC sustained significantly lower JEV replication throughout the examination period compared to wild-type BMDC infected with JEV (1.0 and 10 MOI) (Fig. 6a). Similarly, BMDM obtained from 4-1BB KO mice also showed less JEV replication (Fig. 6b). In support of these findings, the inhibition of JEV replication in 4-1BB-deficient BMDC and BMDM was closely associated with potently enhanced expression of IFN-I (IFN-β) following JEV infection (Fig. 6c, d). BMDC obtained from 4-1BB KO mice showed rapid induction of IFN-β with a 30- to 40-fold increase in response to JEV infection compared to wild-type BMDC. Rapid and increased induction of TNF-α mRNA in 4-1BB-deficient BMDC and BMDM was also observed following JEV infection. Thus, it is likely that potent IFN-I innate responses in myeloid cells derived from 4-1BB KO mice may contribute to the early control of viral replication in the ablation of 4-1BB signaling.

To further characterize enhanced IFN-I innate immune responses in 4-1BB-deficient myeloid cells after JEV infection, we measured the induction levels of antiviral ISG genes. We specifically focused on PRRs (RIG-I [DDX1], MDA5 [IFITH1]) and their transcription factors (IRF3, IRF7), as well as several ISG genes (ISG49 [IFIT3], ISG54 [IFIT2], ISG56 [IFIT1]). 4-1BB-deficient BMDC and BMDM showed differential responses of antiviral ISG expression upon JEV infection. The delayed induction of RIG-I was observed in both 4-1BB-deficient BMDC and BMDM, whereas MDA5 showed rapid induction in 4-1BB-deficient BMDC at 24 h after JEV infection, compared to BMDC derived from BL/6 mice (Fig. 6e). IRF3 was likely not involved in the induction of IFN-I innate responses, because IRF3 expression decreased or was not altered in JEV-infected BMDC and BMDM derived from 4-1BB-ablated mice. The expression of IRF7 was markedly higher in 4-1BB-deficient BMDC and BMDM with a delayed pattern 48 h after JEV infection. In addition, surprising data was obtained from the induction of ISG genes in both 4-1BB-deficient BMDC and BMDM upon JEV infection (Fig. 6f). BMDC and BMDM derived from 4-1BB KO mice showed rapid 2- to 3-fold increases in ISG genes (ISG49, ISG54, ISG56) in response to JEV infection compared to those from BL/6 mice. Collectively, these results indicate that blocking 4-1BB signaling could stimulate rapid and increased IFN-I innate immunity responses in myeloid-derived cells upon JEV infection via induction of antiviral ISG genes, thereby ameliorating JE progression by early control of viral replication.

### Induction of IFN-I and ISGs in 4-1BB signal-deficient primary cortical neurons is coupled to reduction of viral replication

**Fig. 7:**
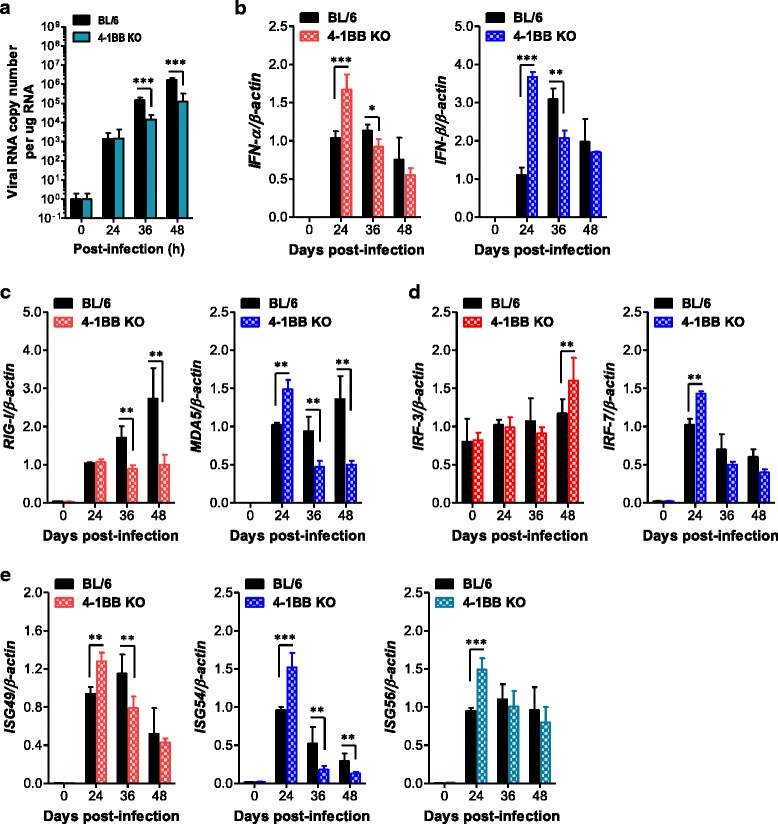
Induction of IFN-I and ISGs in primary cortical neurons from 4-1BB signal-ablated mice after JEV infection. Primary cortical neurons generated from WT and 4-1BB KO mice were infected at MOI of 0.1, and viral replication and IFN-I responses were analyzed by real-time qRT-PCR at 24, 36, and 48 h pi. **a** JEV replication. **b** IFN-I expression. **c**–**e** Induction of RLRs, IRFs, and ISGs. **c** ISGs. **d** RLRs. **e** IRFs. Bar charts show the average ± SD of values derived from primary cortical neurons in quadruplicate. *p < 0.05; **p < 0.01; ***p < 0.001 compared with the levels of the indicated groups

Neurons are the main target cell of JEV infection within the CNS, and their death is a key factor in pathogenesis and neurological sequelae [7]. Furthermore, neuron cells have been recognized to produce antiviral IFN-I in response to viral infection, helping to control viral replication [63, 64]. Accordingly, we examined viral replication and IFN-I innate immune responses in primary cortical neuron cells generated from wild-type BL/6 and 4-1BB KO mice after JEV infection. Consistent with the results derived from myeloid-derived cells, primary cortical neurons derived from 4-1BB KO mice showed reduced JEV replication, even though neuron cells exhibited delayed control of viral replication compared to myeloid-derived cells (Fig. 7a). This reduction of JEV replication in 4-1BB KO neurons was associated with early and increased induction of IFN-I (IFN-α/β) (Fig. 7b). In addition, the expression of antiviral ISGs in 4-1BB-deficient neurons seemed to follow IFN-I innate responses and the reduction of viral replication. Hence, the expression of PRRs (MDA5) and transcription factors (IRF3, IRF7) was observed at higher levels in 4-1BB KO neurons compared to wild-type BL/6 neurons (Fig. 7c, d), even though 4-1BB KO neurons showed moderately different expression patterns of PRRs and transcription factors from those of myeloid-derived cells. Further, 4-1BB KO neurons displayed transiently higher expression of antiviral ISG genes (ISG49, ISG54, ISG56) at 24 h pi, but lower levels of ISG49 and ISG54 in 4-1BB KO neurons were observed 36 h pi compared to wild type (Fig. 7e). Collectively, these results suggest that the ablation of 4-1BB signaling could enhance IFN-I innate immune responses in neuron cells to regulate the spread of JEV in the CNS.

### Dominant role of 4-1BB signal-ablated HSCs in ameliorating JE

**Fig. 8:**
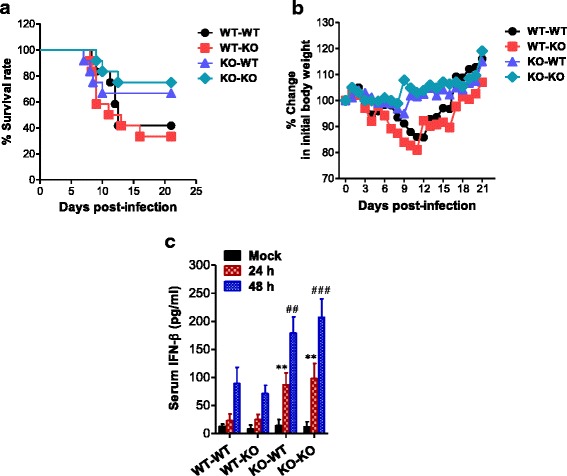
Dominant role of HSCs derived from 4-1BB signal-ablated mice in ameliorating JE. BM cells from WT or 4-1BB KO mice were grafted to lethally irradiated WT or 4-1BB KO recipient mice, which were infected with JEV (3.0 × 10^7^ PFU). **a** Susceptibility of 4-1BB KO BM chimeric models to JE. Infected recipient mice (*n* = 12) were examined over 21 days to determine survival rate. **b** Changes in body weight. Changes in body weight are expressed as the average percentage ± SD of body weight relative to the time of challenge. **c** Systemic IFN-β levels in 4-1BB KO BM chimeras. The amount of serum IFN-β was determined by ELISA at the indicated time points. Bar charts denote the average ± SD of values derived from at least five BM chimeras per group. **p < 0.01 compared with the levels of WT-KO BM chimera at 24 h pi. ^##^ *p* < 0.01; ^###^ *p* < 0.001 compared with the levels of WT-KO BM chimera at 48 h pi

Our results support that 4-1BB signal blockage ameliorates JE progression by providing potent antiviral IFN-I in myeloid-derived cells and early CNS infiltration of mature Ly-6C^hi^ monocytes. In addition, blocking the 4-1BB signal pathway facilitates the development of antiviral NK and Th1 CD4^+^ cells, which may contribute to early control of viral replication. 4-1BB is believed to be expressed primarily on activated T cells and NK cells. However, 4-1BB is also expressed by various immune cells, including neutrophils, monocytes, and macrophages [42–46]. Furthermore, expression of human 4-1BB receptor/4-1BB ligand is not restricted to immune cells, and their functions are more complex than those of mice [41]. Therefore, we sought to test which cell types, focusing on resident cells and myeloid cells derived from hematopoietic stem cells (HSCs), are dominant in the regulation of JE progression in 4-1BB-ablated mice. To this end, we used BM chimeric models of wild-type BL/6 and 4-1BB KO mice. Interestingly, myeloid cells derived from HSCs played a dominant role in conferring amelioration of JE in a 4-1BB-ablated environment, because wild-type BL/6 recipients of 4-1BB KO BM donor cells (KO-WT) showed enhanced resistance to JE, compared to 4-1BB KO recipient of wild-type BL/6 BM donors (WT-KO) and wild-type BL/6 recipients of wild-type BL/6 BM donors (WT-WT) (Fig. 8a). In addition, KO-WT and KO-KO BM chimeric models experienced less change in body weight after JEV infection compared to other BM chimeric models (Fig. 8b). Supporting these findings, potent and rapid IFN-I innate immune responses were observed in the KO-WT BM chimeric model compared to the WT-KO BM chimeric model, based on serum IFN-β levels (Fig. 8c). This result indicates that myeloid cells derived from 4-1BB KO HSCs play a dominant role in IFN-I innate response in 4-1BB KO hosts. Collectively, it appears that blocking 4-1BB signaling in myeloid cells derived from HSCs plays an important role in ameliorating JE by inducing potent and rapid IFN-I innate immune responses.

## Discussion

The impact of the 4-1BB/4-1BBL co-stimulatory pathway on antiviral immunity has been studied in several viral infection models using gene knockout systems. Although 4-1BB/4-1BBL interactions result in both positive and negative impacts on viral infection depending on the type of virus, disease severity, and timing of 4-1BB signal blockade [47–54], our data demonstrate that blocking the 4-1BB signaling pathway provides increased resistance to JE, rather than causing detrimental effects. This finding is supported by our observation that treatment with a 4-1BB agonistic mAb (3E1) exacerbated JE. The reduction of viral burden in extraneural tissues and the CNS by blocking the 4-1BB signal pathway correlated with an increased frequency of IFN-γ-producing NK and CD4^+^ Th1 T cells as well as increased accumulation of mature Ly-6C^hi^ monocytes in the inflamed CNS. More interestingly, DCs and macrophages derived from 4-1BB KO mice showed potent and rapid IFN-I innate immune responses in response to JEV infection, which could represent inhibition of JEV replication. In addition, 4-1BB signal-ablated neuron cells displayed enhanced IFN-I innate responses to JEV infection compared to normal neuron cells. Ultimately, these results imply that the promotion of IFN-I/II responses in a 4-1BB signal-ablated environment contribute to the inhibition of viral replication at the periphery and CNS, thereby ameliorating JE progression. Therefore, our data suggest that regulation of the 4-1BB signaling pathway with blocking mAb or inhibitors could represent a valuable therapeutic target in the treatment of JE.

The role of IFN-γ, the only member of IFN-II, is rather unclear in immune-mediated protection against viral disease of the CNS [65]. In particular, the requirement of IFN-γ in recovery from infections with different flaviviruses has been shown vary. IFN-γ plays a crucial role in early protective immune responses against a virulent North American isolate of WNV [23] and mouse-adapted strains of dengue virus [24, 25], but is dispensable in the control of infection with less virulent strains of WNV [26] or yellow fever virus [27, 28], and shows only a modest protective role against Murray Valley encephalitis [29]. Similarly, IL-12 has been reported to show suppressed protective immunity to JEV in mice through IFN-γ [66], but in some experiments, IFN-γ was associated with a beneficial effect on the outcome of JE [22]. Our data favor the latter results that show a beneficial role of IFN-γ in JE progression. IFN-γ is involved in diverse functions for the control of microbial infections, including activation and polarization of CD4^+^ Th cells, upregulation of Fas in infected target cells, upregulation of MHC I- and II-restricted Ag-presentation pathways, macrophage activation, and direct antiviral activity that overlaps with activities triggered by IFN-I [67]. Conceivably, it is possible that enhanced production of IFN-γ by NK and polyclonal CD4^+^ Th1 T cells in a 4-1BB-blocked environment is involved in the early control of viral replication in extraneural tissues and the CNS. However, considering that NK cell-depleted mice show no change in viral burden or survival [29], NK cell responses do not appear to significantly contribute to host survival, even though infection with JEV provides early activation of NK cells. This notion is consistent with the absence of a protective value of NK cells against WNV [68]. Furthermore, flaviviruses, including WNV, exhibit immune escape from NK-cell attack involving the upregulation of MHC-I in infected cells [69]. Therefore, it is unlikely that IFN-γ produced from NK cells and the cytolytic function of NK cells via granzyme B is dominant in the regulation of JE progression.

The cytolytic function of infected target cells by antigen-specific CD8^+^ T cells is thought to play a crucial role in disease recovery, given that depletion of CD8^+^ T cells results in increased viral burden in the CNS [70]. In addition, CD4^+^ T cells that show IFN-γ-producing Th1 type in response to JEV Ag appear to elicit an important protective immune parameter for the control of JEV [21]. Because of impaired and delayed JEV-specific CD4^+^ and CD8^+^ T cell responses in 4-1BB signal-ablated mice, our data suggest that IFN-γ-producing CD4^+^ Th1 cells may be dominant players in the control of viral replication during the early phase. In addition, this fact raises the notion that the equilibrium of IFN-γ-producing CD4^+^ Th1 and IL-17-producing CD4^+^ Th17 cells may become an important parameter for prognosis in JE progression. Here, one interesting result was that the frequency or number of CD4^+^CD25^+^Foxp3^+^ Treg cells was not apparently changed by the ablation of 4-1BB signal. Because an increased number of CD4^+^CD25^+^Foxp3^+^ Treg cells is correlated with milder forms of encephalitis caused by flavivirus infection [58], this finding suggests that IFN-γ produced from CD4^+^ Th1 cells can affect the progression of JE without changing the number of CD4^+^CD25^+^Foxp3^+^ Treg cells.

IFN-γ produced by CD4^+^ Th1 cells appears to be involved in the maturation of myeloid-derived cells, including Ly-6C^hi^ monocytes [71, 72]. Further, IFN-I produced by myeloid-derived cells, including DCs and macrophages, is likely to play an important role in the differentiation and function of Ly-6C^hi^ monocytes [73]. These notions support the increased accumulation of mature CD11b^+^Ly-6C^hi^ monocytes in both inflamed CNS and lymphoid tissues of 4-1BB KO mice via enhanced responses of IFN-II-producing NK and CD4^+^ Th1 cells and IFN-I innate responses in 4-1BB-deficient myeloid cells. In addition, activated microglia/macrophages in the CNS of 4-1BB KO mice showed higher expression levels of MHC II molecules that could be induced by IFN-γ produced from NK and CD4^+^ Th1 cells [61, 62]. Therefore, it is likely that mature CD11b^+^Ly-6C^hi^ monocytes infiltrated into the CNS of 4-1BB KO mice exert a more effective regulatory effect in JE progression compared to those of wild-type BL/6 mice. To date, the roles of CD11b^+^Ly-6C^hi^ monocytes in CNS inflammation caused by neurotropic viruses have not been clearly delineated due to conflicting results [33–36]. CD11b^+^Ly-6C^hi^ monocytes cause significant damage and destruction that exacerbate morbidity and mortality [74, 75], whereas CNS infiltration of CD11b^+^Ly-6C^hi^ monocytes plays a protective role during CNS inflammation [33–36]. Because the enhanced infiltration of CD11b^+^Ly-6C^hi^ monocytes in the CNS of 4-1BB KO mice correlates with better survival, our data support their beneficial role in JE progression. Although the detailed mechanisms by which infiltrated CD11b^+^Ly-6C^hi^ monocytes regulate neuroinflammation caused by neurotropic viruses remain to be defined, it is thought that CD11b^+^Ly-6C^hi^ monocytes exert regulatory functions through differentiation into DCs, macrophages, and microglia [30–32]. In support, we found that the mature macrophage marker F4/80 was expressed in CD11b^+^Ly-6C^hi^ monocytes of 4-1BB KO mice at much higher levels than in wild-type BL/6 mice. In addition, ablation of the 4-1BB/4-1BBL system may enhance the anti-pathogen response by induction of myelopoiesis, resulting in the generation of more myeloid cells that can enhance the strength and efficiency of anti-pathogenic immune responses [76–78]. CD11b^+^Ly-6C^hi^ monocytes are derived from BM, travel through blood and subsequently arrive at inflamed tissues, depending on expression of the CCR2 chemokine receptor [30]. Thus, it is possible that CD11b^+^Ly-6C^hi^ monocytes in a 4-1BB signal-ablated environment may be in greater supply in the CNS during JE progression.

The most intriguing result in this study was that DCs and macrophages derived from BM cells of 4-1BB-ablated mice showed potent and rapid IFN-I innate immune responses to JEV infection. This presumably promotes early clearance of the virus at the periphery, because DCs and macrophages are primary target cells for JEV infection. Although the detailed mechanisms behind enhanced IFN-I responses in DCs and macrophages derived from 4-1BB KO mice are not defined, our data suggest that the enhanced stimulation of intracellular PRRs (RIG-I, MDA5) and subsequent activation of their transcription factors (IRF7) may be involved in potent IFN-I innate immune responses in 4-1BB-deficient DCs and macrophages. In addition, the potent IFN-I response of DCs and macrophages derived from 4-1BB KO mice may be indirectly mediated by soluble factors produced from host cells by viral infection, i.e., DMAPs. We did not exclude the potential interaction of the 4-1BB signaling pathway with pathways of PRRs that recognize JEV infection in myeloid-derived cells. Considering that only a small fraction (10–20 %) of myeloid-derived cells are infected by JEV [79], uninfected myeloid-derived cells are thought to contribute substantially to antiviral ISG induction through stimulation of IFNAR and their transcription factor STAT1, thereby inducing ISG49, ISG54, and ISG56 [80]. In addition, somewhat interestingly, neuron cells derived from 4-1BB KO mice exerted increased IFN-I innate responses. However, different induction patterns of PRRs and their transcription factors between JEV-infected neuron and myeloid-derived cells indicate that specific types of cells differentially trigger IFN-I innate immune responses following JEV infection. Ultimately, despite the clear induction of potent and rapid IFN-I innate immune responses in 4-1BB-deficient myeloid cells and neurons, future studies will be required to delineate the mechanistic and functional intermediates that link and regulate IFN-I innate immune responses in the absence of the 4-1BB signaling pathway.

JE pathogenesis in the murine model may be altered by the route of administration, virus propagation conditions, or strain of virus [11]. Although JEV infection via an i.p. route may not directly reflect natural infection mediated by the intradermal or subcutaneous route taken when an organism is bitten by mosquitoes, JEV introduced via an i.p. route shows entirely similar pathogenesis to a natural infection, due to peripheral amplification in the spleen. Furthermore, mice infected with JEV usually display a neurological disorder at 4–5 dpi. Thus, rapid innate immune responses, including IFN-I of myeloid cells and IFN-II of NK and CD4^+^ Th1 cells, are more critical in controlling JE progression compared to delayed Ag-specific adaptive responses.

## Conclusions

Blocking the 4-1BB signaling pathway ameliorates JE via divergent enhancement of IFN-II-producing NK and CD4^+^ Th1 cells and mature Ly-6C^hi^ monocyte infiltration, as well as the IFN-I innate response of myeloid-derived cells. Therefore, blocking the 4-1BB signaling pathway with antagonistic mAb or inhibitors may be a valuable therapeutic tool for the control of JE progression via enhanced IFN-I and IFN-II responses.

## Abbreviations

BBB: blood–brain barrier
BM: bone marrow
BMDC: bone marrow-derived dendritic cell
BMDM: bone marrow-derived macrophage
dpi: days post-infection
HSC: hematopoietic stem cell
IFN-I/II: type I/II interferon
JE: Japanese encephalitis
KO: knockout
TLR: Toll-like receptor
TNFR: tumor necrosis factor receptor
